# Internal and external signal processing in patients with panic disorder: An event-related potential (ERP) study

**DOI:** 10.1371/journal.pone.0208257

**Published:** 2018-11-29

**Authors:** Christian Valt, Dorothea Huber, Ingrid Erhardt, Birgit Stürmer

**Affiliations:** 1 International Psychoanalytic University Berlin, Berlin, Germany; 2 Department of Psychosomatic Medicine and Psychotherapy, Klinikum München, Munich, Germany; Universita degli Studi di Padova, ITALY

## Abstract

Self-absorption describes a pathological tendency towards the internal mental world (internalization) that often conflicts with the accurate monitoring of the external world. In performance monitoring, an augmented electrophysiological response evoked by internal signals in patients with anxiety or depressive disorder seems to reflect this tendency. Specifically, the error-related negativity (Ne/ERN), an index of error processing based on internal signals, is larger in patients compared to controls. In the present experiment, we investigated whether the preferential processing of internal signals in patients is linked to diminished and inflexible external signal processing. To this end, the electrophysiological response evoked by external signals was analysed in patients with panic disorder and healthy controls. Participants performed a choice-response task, where informative or uninformative feedback followed each response, and a passive viewing task. As a replication of previous studies, patients presented an augmented Ne/ERN, indexing enhanced processing of internal signals related to errors. Furthermore, the vertex positive potential (VPP) evoked by visual stimuli was larger in patients than in controls, suggesting enhanced attention to external signals. Moreover, patients and controls showed similar sensitivity to the feedback information content, indicating a normal flexibility in the allocation of monitoring resources to external signals depending on how informative these signals are for performance monitoring. These results suggest that the tendency towards internal signals in patients with panic disorder does not hinder the flexible processing of external signals. On the contrary, external signals seem to attract enhanced processing in patients compared to controls.

## Introduction

A balanced processing of the internal mental world and the external world is a key aspect for everyday well-being. In fact, a pathological tendency towards the internal world (internalization) is a common factor in psychological disorders characterised by anxiety, depression, and obsessive-compulsive symptoms [1, 2]. Although internal attention is often appropriate, many situations require stronger attention to information from external sources; consequently, a rigid, excessive and sustained self-focused attention could hamper a realistic evaluation of one-self based on relevant environmental signals. Ingram [3] described the inflexible focus towards internal signals with the concept of self-absorption. With the present study, we employed electrophysiological recordings in an experimental context of performance monitoring to test whether patients with a psychological disorder characterised by internalization (i.e. panic disorder) present a deficit in the flexible processing of internal and external signals and whether the tendency towards internal signals hinders the accurate processing of external signals. To this end, we employed an experimental design where participants could evaluate their performance in a response-choice task based on self-generated signals or feedback. This experiment enabled the analysis of potential differences in the flexible and accurate processing of internal and external signals between patients and controls as modulations of the error-related negativity (Ne/ERN; [4, 5]), an index of internal monitoring, and the N170/VPP complex [6], an index of attention on external visual stimuli [7].

In electrophysiological studies of performance monitoring, enlarged potentials associated with the evaluation of response accuracy based on internal signals seem to reflect the pathological tendency towards the internal world in psychological disorders characterised by internalization [8]. In fact, studies of generalised anxiety disorder (GAD), major depressive disorder (MDD) and obsessive-compulsive disorder (OCD) have repeatedly reported the presence of a larger error-related negativity (Ne/ERN) in patients compared to controls (for reviews, see [9–11]). The Ne/ERN is an event-related potential (ERP) evoked immediately after a response and reflects error detection according to the comparison of self-generated signals associated with the performed action and the desired action plan. The link between internalization psychopathology and enhanced Ne/ERN is so tight that researchers consider the abnormal processing of internal signals evoked by errors as an endophenotype of internalization disorders [8, 12].

At present, electrophysiological results on performance monitoring are in line with the interpretation of hyperactive monitoring of internal signals in internalization disorders. However, the investigation of the consequent inference of an abnormal processing of external signals is limited to few studies, and the pattern of results is mixed. For example, research on feedback processing in clinical or subclinical populations of people with symptoms of depression observed, in some studies, reduced [13–18] and, in other studies, enhanced feedback processing [19–22]. As reported by Moran and collaborators [10], the observation of different patterns of feedback processing across experiments might depend on the task. In fact, reduced feedback processing was observed in gambling tasks, whereas enhanced processing occurred in tasks where feedback was contingent on performance, as in time-estimation or reinforcement learning tasks. Different patterns of activity across tasks might, therefore, depend on the presence or the absence of a link between performance and outcome, affecting the feeling of control over the outcome [23] and the dynamic of feedback processing [24]. Unfortunately, the picture of external signal processing in psychological disorders characterised by internalization does not get clearer in studies on obsessive-compulsive disorder [9, 25–28] or anxiety disorder [29–31]. Consequently, the large variability in the electrophysiological results across studies on feedback processing in GAD, MDD and OCD does not allow to advance conclusions on whether internalization reduces the processing of external signals. Moreover, in a recent study, Burkhouse and collaborators [32] found that feedback processing in internalizing psychopathologies might differ according to the level of distress/misery or fear symptoms [1]. In fact, abnormal feedback processing seems to be linked to the severity of distress/misery but not to fear symptoms.

Importantly, studies on external signal processing in GAD, MDD, or OCD focused on the feedback-related negativity (FRN). This ERP is thought to be the feedback-locked counterpart of the Ne/ERN [33, 34], and it reflects the processing of an external signal for performance monitoring. Similar to the Ne/ERN, this component is more negative when feedback indicates the commission of an error or an unfavourable outcome compared to when it describes a correct and favourable performance [35]. This ERP reflects feedback evaluation according to valence [36, 37], expectancy [38, 39], or information content [24, 40–42]. Consequently, studies on the effects of internalization on the FRN might indicate an abnormal feedback evaluation but they do not directly clarify whether a focus towards the internal world distracts attentional resources from the accurate and flexible processing of the external world.

In a recent study conducted in our lab, we observed that informing participants about a link between personal performance and emotional faces presented after each response enhanced the amplitude of the N170 and reduced the Ne/ERN [43]. The N170 is an early visual potential evoked at parieto-occipital electrodes by the structural encoding of faces [44]. Outside the context of performance monitoring, studies on the effects of attention on face perception showed that the allocation of attention on a face stimulus leads to enhancements of the N170 [7]. Therefore, the N170 and its medial fronto-central counterpart [6]—the vertex positive potential (VPP; [45])—might represent an electrophysiological marker of attention suitable for the investigation of external signal processing in psychological disorders.

The present experiment investigated whether a focus towards the internal world in a psychological disorder characterised by internalization implies a reduced and inflexible processing of the external world. To this end, we analysed the brain response evoked by feedback in a highly homogenous group of patients with panic disorder and comorbidity of a personality disorder and a group of healthy controls. The DSM-5 classifies the panic disorder as an anxiety disorder, characterised by unexpected and recurrent panic attacks. Contrary to phobias, panic attacks (like palpitations, sweating, trembling, and others) in panic disorder are not specific to a particular external stimulus or situation but they seem general and spontaneous. Similar to other anxiety disorders, panic disorder is a psychopathology characterised by internalization and self-focused attention, but it is distinguishable from GAD, MDD, and OCD because of a higher level of fear than misery/distress symptoms [1, 46].

In this experiment, patients and controls had to perform a response-choice task with feedback presented after each response. Throughout the experiment, feedback was informative about the personal performance, in half of the experimental blocks, and uninformative, in the other experimental blocks. Changing the feedback information content should induce different processing of external signals because only informative feedback was meaningful for performance monitoring. If internalization interferes with the flexible processing of external signals according to their information content, patients should present significant differences from controls in their pattern of brain activity evoked by external signals in the two different feedback conditions. Previous studies showed that early feedback-locked ERPs are smaller when the feedback is uninformative about performance compared to when it is informative [24, 47], suggesting that the monitoring system can flexibly allocate attentional resources to external signals according to the feedback information content. Therefore, a deficit of flexibility in the patient group should result in an insensitivity of the feedback-locked ERPs to the feedback manipulation across experimental blocks. We did not expect effects of the feedback information content on the internal signal processing because Olvet and Hajcak [48] showed that presenting an informative feedback in a response-choice task does not reduce the amplitude of the response-locked ERPs, suggesting that a focus on external signals does not subtract monitoring resources from internal signal processing. Global differences between patients and controls, independent of the feedback information content, would instead highlight a general deficit in the processing of external signals in patients with panic disorder. According to self-absorption, a pathological focus towards the internal world should lead to reduced attention to the external world, with a consequent smaller amplitude of the ERPs related to attention on external visual stimuli, like the N170/VPP. Joyce and Rossion [6] reported that the N170 and the VPP reflect the activity of the same neural generator. This activity is mainly evident as N170 when the signal is referenced to the average scalp activity or as VPP when the signal is referenced to activity recorded by the electrodes placed over the mastoids. The described link between VPP and N170 is highly relevant for the study of the processing of external signals for performance monitoring because some experiments observed that different experimental manipulations induce significant effects on the positive potential that peaks before the FRN [49, 50]. For example, in Valt, Sprengeler and Stürmer [24], feedback descriptive of the personal performance evoked a potential with a larger positivity before the FRN than the one elicited by random feedback, suggesting a larger interest for informative compared to uninformative feedback. Based on the similarities in topographical and temporal properties between the VPP evoked by faces and the positive peak evoked by external signals (coloured squares in [24]), it is reasonable to speculate that these two potentials describe the same process. Therefore, in the present experiment, patients should present enhanced Ne/ERN elicited by errors and reduced VPP or FRN evoked by feedback.

To qualify the relevance of a context of performance monitoring for the manifestation of potential abnormal processing of external signals, we incorporated in the experiment a passive viewing task with pictures of faces or houses. The presence of different processing of the stimuli also in this task would indicate that the abnormal brain responses evoked by external signals are not restricted to the context of performance monitoring but generalise to other perceptual tasks.

To summarize, we investigated whether, within a context of performance monitoring, a focus towards the processing of internal signals in panic disorder, that should be evident as augmented Ne/ERN, leads to a reduced and inflexible processing of external signals (e.g. feedback). We hypothesised that patients and controls should present significant differences in the brain response evoked by external signals. Compared to controls, the VPP and the FRN in patients should be smaller and insensitive to the feedback information content.

## Methods and materials

### Participants

Twenty-two patients suffering from panic disorder with a comorbidity of a personality disorder (10 female) and twenty-two healthy age-, gender-, education-matched control participants (10 female) took part in the study. The mean age of patients was 38 years (*SE*: 2.44; age range: 20–55) and control participants had a mean age of 38 years (*SE*: 2.57; age range: 19–58).

Patients were recruited in cooperation with a study on the efficacy of cognitive-behavioural and psychoanalytic therapy in panic disorder with a comorbidity of a personality disorder [51] funded by the German Society for Psychoanalysis, Psychotherapy, Psychosomatic Medicine and Depth Psychology (Deutsche Gesellschaft für Psychoanalyse, Psychotherapie, Psychosomatik und Tiefenpsychologie, DGPT). Patients matching the required psychological profile were selected based on the German version of the Structured Clinical Interview for DSM-4 (SCID, [52]) and they were invited to take part in this EEG study before the beginning of therapy. Patients were recruited at the Department of Psychosomatic Medicine and Psychotherapy, Klinikum München (N = 12), or at the psychotherapy ambulance of the International Psychoanalytic University Berlin (N = 10). Participants for the control group were recruited from the local community in Berlin through announcements on the web, with the restriction of no history of neurological or psychological disorders. The experiment was conducted in Munich at the Department of Psychosomatic Medicine and Psychotherapy, Klinikum München, and in Berlin at the International Psychoanalytic University Berlin.

All the patients had a diagnosis of panic disorder. Fourteen patients had also agoraphobia and all the patients had at least one personality disorder categorised in the Cluster C of the DSM-5 (anxious/fear: avoidant, dependent, obsessive-compulsive) or a depressive personality disorder according to the DSM-4. Patients with eating, addictive, psychotic, or bipolar disorder were not included in the study.

Table 1 provides the average cumulative scores of both groups in the German versions [53–55] of the Penn State Worry Questionnaire (PSWQ, [56]), the trait subscale of the State-Trait Anxiety Inventory form Y2 (STAI-t, [57]), and the Beck Depression Inventory (BDI, [58]). The Cronbach’s alphas for the questionnaires in patients with panic disorder were 0.91 for the BDI, 0.87 for the PSWQ, and 0.65 for the STAI-t, which indicate a high level of internal consistency for the three scales with the sample of patients tested in this experiment. Patients suffering from panic disorder and a personality disorder reported higher symptoms severity of anxiety (STAI-t), *t*(42) = 4.55, *p* < .001, worry (PSWQ), *t*(42) = 5.86, *p* < .001, and depression (BDI), *t*(42) = 6.06, *p* < .001.

**Table 1:** Mean scores in the questionnaires.

	Patients	Controls	
**PSWQ**	56.86 (2.19)	38.68 (2.20)	*p* < .001
**STAI-t**	47.77 (1.13)	41.36 (0.85)	*p* < .001
**BDI**	24.32 (2.31)	6.93 (1.70)	*p* < .001

PSWQ: Penn State Worry Questionnaire; STAI-t: trait subscale of State-Trait Anxiety Inventory; BDI: Beck Depression Inventory

All participants had a normal or corrected-to-normal vision and, according to the Edinburgh Handedness Inventory [59], they were right-handed, except for three left-handed and two ambidextrous participants in the patients group, and three left-handed participants in the control group. The ethics committee at the International Psychoanalytic University Berlin approved the study (protocol 2015–1) and participants gave their written informed consent before the beginning of the experiment. Participants received a monetary compensation of 20 € for their participation in the study.

### Task and procedure

The experiment consisted of a response-choice task followed by a passive viewing task. The response-choice task started with three practice blocks where participants familiarised with the task and the relationship between performance and feedback. The experiment proper had twenty experimental blocks, divided into four separate runs. The passive viewing task was a sequence of pictures of faces or houses with a break after half of the stimuli. The response-choice task lasted for around 45 minutes, whereas the duration of the passive viewing task was around 7 minutes.

At the end of the experiment, participants filled the Penn State Worry Questionnaire, the trait subscale of the State-Trait Anxiety Inventory form Y2, and the Beck Depression Inventory to evaluate the presence and severity of depression and anxiety symptoms.

#### Response-choice task

**Fig 1:**
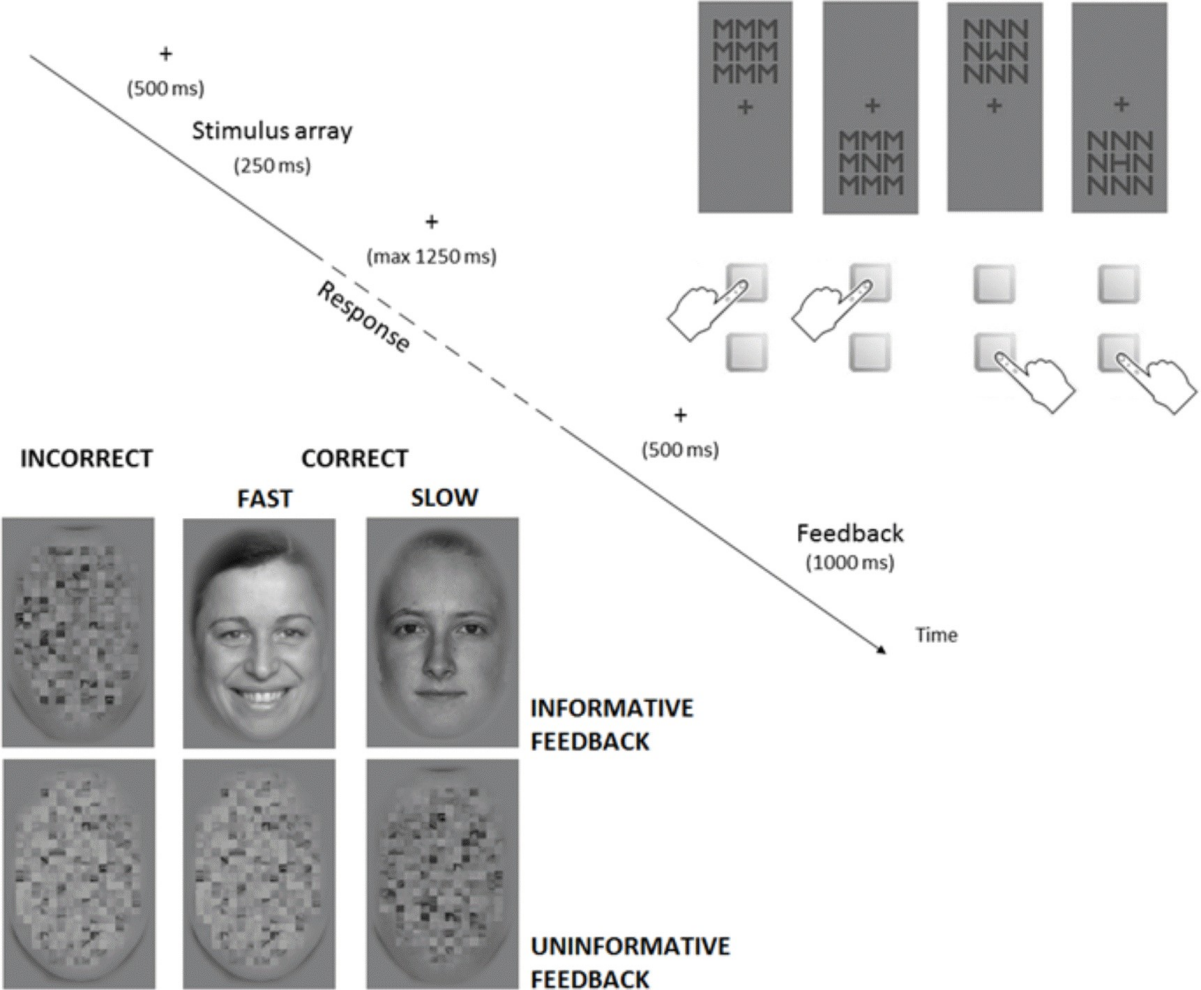
Design of the response-choice task. Schematic representation of the response-choice task, the time course of trials, and the stimuli used as external signals in the conditions with informative and uninformative feedback.

In the response-choice task, participants had to make a button press in reaction to the identity of the central letter in a 3x3 array of the letters M, N, W, or H, arranged as eight identical letters framing a central letter (target). Two response buttons, placed vertically on the desk, had to be used according to an instructed stimulus-response mapping that linked two letters (e.g. M and N) to one button and the other two letters (e.g. W and H) to the other button. Letter arrays could appear above or below the fixation cross. The incongruency between the response evoked by the target letter and the response associated with the flanker letter determined an Eriksen conflict [60], whereas the incongruency between the position of the letter array on screen and the relative position of the buttons on the desk induced a Simon conflict [61]. Fig 1 depicts an example of letter arrays incorporating different levels of response conflict.

Within a trial, feedback was delivered after each response or at the end of the response period if no button had been pressed (see Fig 1). In half of the experimental blocks, feedback was always a scrambled face, irrespective of response accuracy and speed (uninformative feedback), whereas, in the other half, feedback precisely reflected response quality (informative feedback). In the condition with informative feedback, a scrambled face appeared only after errors or missing responses, while faces with a neutral or a happy expression were feedback, respectively, of correct slow and correct fast responses. Response speed (fast or slow) was determined according to the median response time (RT) in the previous twenty-three trials with correct responses. Feedback condition changed after five experimental blocks, producing an alternation between runs with informative feedback and runs with uninformative feedback. Changes of the feedback condition were signalled via instruction before the beginning of each run. The stimulus-response mapping and the sequence of runs with informative or uninformative feedback were counterbalanced across participants.

Feedback stimuli were 170 pictures of neutral faces and 170 pictures of happy faces from the stimuli set FACES [62], and 540 similar faces scrambled with PhotoShop. All the stimuli were converted to grey scale and fitted in a rectangular shape with rounded edges (size 6.81° x 4.52° of visual angle).

The parametric combination of four target letters, four flanker letters, and two array positions generated 32 different trials, performed once within each experimental block. Each trial started with the presentation of the stimulus array, displayed on the screen for 250 ms, followed by a response period with a self-paced duration of maximum 1,250 ms. Feedback was presented for a duration of 1,000 ms after the end of the response period or 500 ms after the response. The new trial started 500 ms after feedback offset. During periods without stimulus array or feedback, only the fixation cross was on screen. The letters in the stimulus array (size of 0.32° x 0.32°, separated by gaps 0.05°) and the fixation cross (size: 0.32° x 0.32°) had a dark grey colour (RGB: 78, 78, 78) and were displayed on a light grey background (RGB: 128, 128, 128). The stimulus arrays could appear above or below the fixation cross (with a centre-to-centre distance of 0.80°).

Throughout the experiment, errors and slow responses were punished by the subtraction, respectively, of 0.05 € and 0.02 € from a starting bonus of 15.00 €. At the end of each run of five experimental blocks, participants were informed about the amount of money left in the bonus, which was then granted at the end of the experiment. This procedure was adopted to invite a constant focus on the task, irrespective of the feedback condition. Moreover, written feedback presented at the end of each run encouraged the participant to be faster or more accurate if the percentage of errors in the run distanced substantially from the ideal error rate of 10%.

#### Passive viewing task

In the passive viewing task, 150 pictures of faces with a neutral expression and 150 pictures of houses were presented randomly in sequence (stimuli for this task were taken from [63]). Stimuli were displayed for 1,000 ms, and they were separated one another by intervals of 500 ms blank screen. Instructions invited the participants to pay attention both to faces and houses, to be able to make an accurate recognition of the stimuli when requested. In fact, as an attentional check, for 1/7 of the pictures, the sequential presentation of stimuli stopped and participants had to perform a 1-back recognition task, reporting whether the stimulus on screen was identical to (50% of the times) or different from (50% of the times) the stimulus presented immediately before. In the case of a recognition error, the participant was requested to pay more attention to the stimuli.

### EEG recording and signal processing

Throughout the whole duration of the experiment, 28 Ag/AgCl electrodes mounted in an elastic cap (Easycap GmbH) and 2 electrodes applied directly on the skin over the left and the right mastoid (M1 and M2) recorded the electroencephalography (EEG). According to the 10/20 System, the location of the electrodes in the cap corresponded to the positions Fp1/2, F7/8, F3/4, Fz, FC3/4, FCz, T7/8, C3/4, Cz, CPz, P7/8, P3/4, Pz, PO7/8, PO9/10, O1/2, Oz. The EEG was referenced to the left mastoid, and the electrode AFz served as the ground. Two electrodes placed on the outer canthi of both eyes (horizontal EOG) and one electrode placed below the right eye (vertical EOG) recorded the electrooculogram (EOG). All signals were digitalised with a frequency of 500 Hz and a band-pass filter between 0.05 and 70 Hz. Electrodes’ impedance was kept below 10 kΩ for all the electrodes.

Offline the influence of blinks, eye-movements, and pulse artefacts was corrected with independent component analysis trained on calibration trials performed at the end of the experiment. The signal was then further filtered with a band-pass of 0.01 to 30 Hz and a slope of 48 dB/octave.

The EEG signal was segmented to create response-locked and feedback-locked epochs in the response-choice task and stimulus-locked epochs in the passive viewing tasks. Epochs started 200 ms before the marker of interest and lasted for 800 ms, when response-locked, or 1200 ms, when feedback- or stimulus-locked. Based on visual inspection, epochs with artefacts were discarder from the analyses. All epochs were re-referenced to the average activity of the mastoid electrodes and aligned to the 200 ms period preceding the 0 point of the epoch.

Epochs related to performance monitoring in the response-choice task (response-locked and feedback-locked) were averaged according to performance (incorrect, correct-fast, and correct-slow) and feedback condition (informative feedback or uninformative feedback). Epochs related to the perceptual processing of stimuli in the passive viewing task were averaged according to the type of stimulus (face or house). The response-related negativity was computed as average activity at electrode Fz in the time-window 0–100 ms of the response-locked ERPs. For the identification of the Ne/ERN component, activity evoked by errors was contrasted against the activity elicited by correct responses. The FRN was computed as average activity at electrode Cz in the time-window 200–300 ms of the feedback-locked ERPs. Since the signal was referenced to the average activity recorded by the mastoid electrodes, the amplitude of the potential evoked by visual stimuli was calculated for the VPP at Cz, instead of the N170 over parieto-occipital electrodes. The VPP was calculated as the peak-to-peak difference between the maximum negative amplitude between 100 and 160 ms and the maximum positive amplitude between 140 and 200 ms after feedback onset. Similarly, the VPP was also computed for the brain response evoked by faces and houses in the passive viewing task.

Behavioural and electrophysiological results were analysed with repeated measures ANOVAs with Group (patients vs. controls) as the between-participants factor. In the response-choice task, the Ne/ERN analysis had only one within-participant factor, Feedback Condition (informative vs. uninformative), whereas the VPP and FRN analysis had two within-participant factors, Feedback Condition (informative vs. uninformative) and Performance (incorrect, correct-fast, vs. correct-slow). In the passive viewing task, Stimulus (faces vs. houses) was the only within-participant factor. For the factor Performance, degrees of freedom were corrected according to Greenhouse-Geisser correction to account for significant violations of sphericity and post-hoc related-samples two-tailed *t*-tests analysed the direction of significant effects or interactions. The significance level of the ANOVAs was α = .05, whereas, in the *t*-tests performed to explore significant effects of Performance, α was adjusted to .016 according to Bonferroni correction to account for multiple tests.

## Results

### Behavioural results

#### Response-choice task

**Fig 2:**
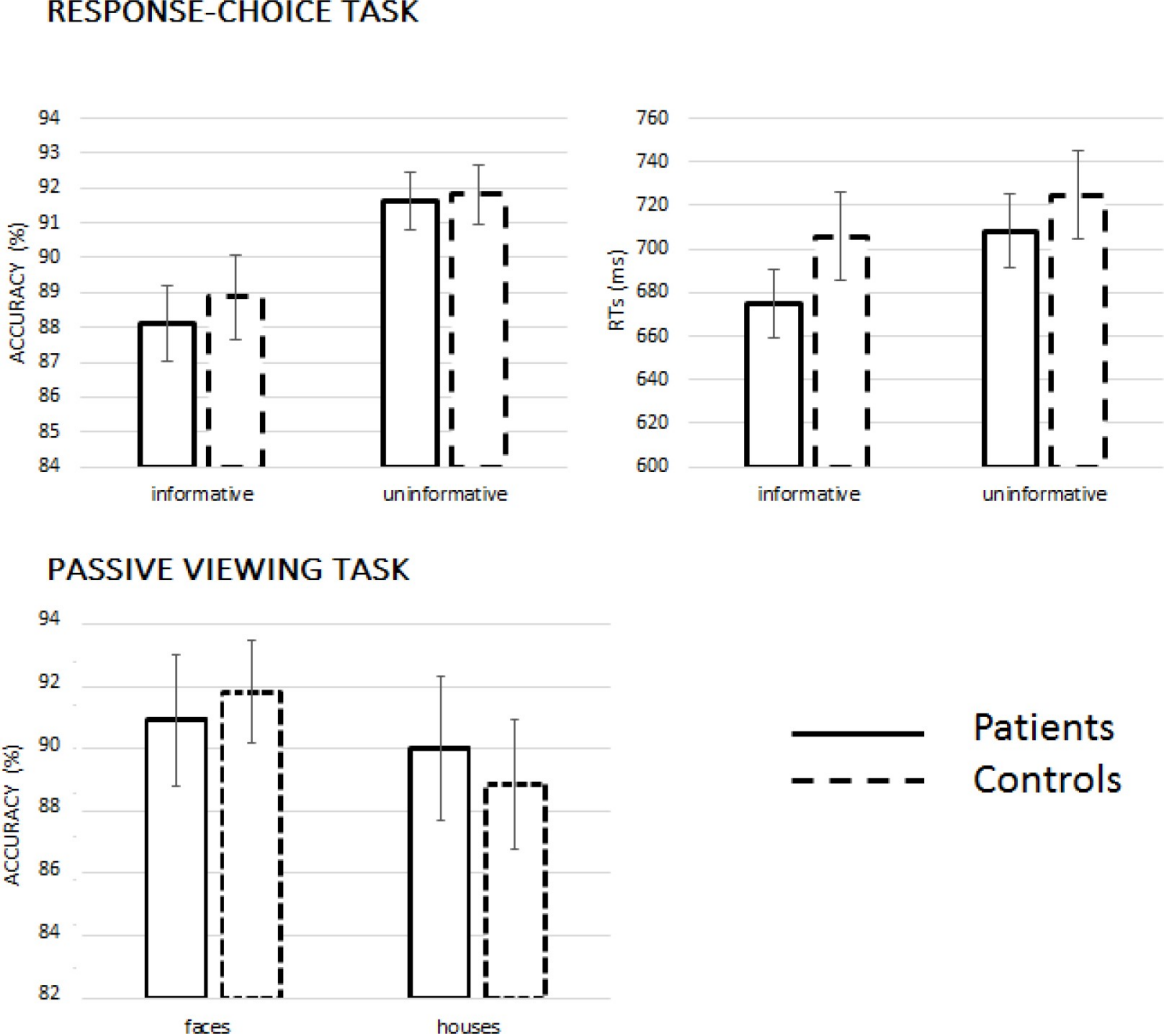
Behavioural results. Mean accuracy and RTs in the experimental tasks.

On average, 0.7% and 0.5% of trials were discarded, respectively, in the patient and in the control group, because of missing responses. The average error rate was 10.1% (*SE* = 0.9%) in the group of patients and 9.6% (*SE* = 1.0%) in the group of control participants. The ANOVAs on accuracy and response times showed significant main effect of Feedback Condition, *F*(1,42) = 45.31, *p* < .001, *η*^*2*^_*p*_ = .519, and , *F*(1,42) = 25.38, *p* < .001, *η*^*2*^_*p*_ = .377, indicating that participants were significantly faster but less accurate in trials with informative feedback compared to trials with uninformative feedback (see Fig 2). However, patients and controls did no present significant differences in their behavioural performance, *F*s < 1.

#### Passive viewing task

Accuracy in the 1-back recognition task was analysed by an ANOVA contrasting the recognition performance for faces and houses between the two groups. Accuracy for faces and houses was not significantly different, *F*(1,42) = 2.05, *p* = .160. Moreover, patients and controls did not present any significant difference in their accuracy in the 1-back recognition task, *F*s < 1 (see Fig 2).

### ERP results

#### Response-choice task

As a precondition for studying whether Group affected the Ne/ERN, we first investigated by an ANOVA whether incorrect responses elicited this ERP at all. The effect of Accuracy (incorrect and correct) on the response-related activity between 0 and 100 ms was significant, *t*(43) = 8.00, *p* < .001, indexing the presence of a larger negativity after errors than after correct responses. Based on this outcome, we can conclude that, within the present design, the Ne/ERN was released in trials with incorrect responses.

**Fig 3:**
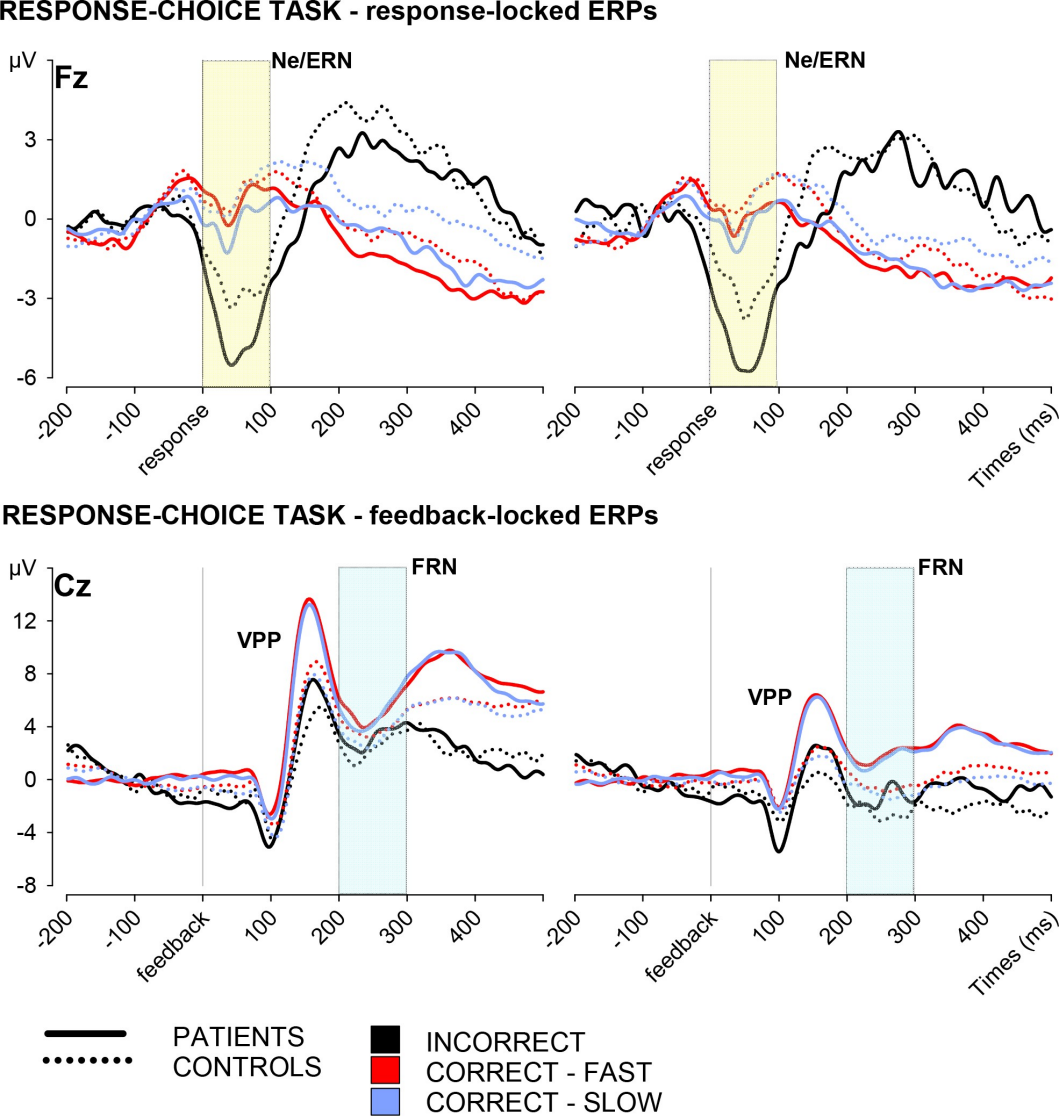
ERP results–response-choice task. Response-locked and feedback-locked grand average ERP waves for the processing of internal and external signals in the response-choice task. Colour areas highlight the time-windows considered for the calculation of the Ne/ERN (blue) and the FRN (pink).

The ANOVA performed on the Ne/ERN evoked by errors in trials with informative or uninformative feedback showed a significant main effect of Group, *F*(1, 42) = 5.46, *p* = .024, *η*^*2*^_*p*_ = .115 (see Fig 3). The Ne/ERN evoked by the processing of errors based on internal signals was larger in patients than controls. The effect of Feedback Condition was not significant, *F* < 1, suggesting that changing the information content of the feedback did not affect the monitoring of internal signals. Moreover, the interaction between Feedback Condition and Group was not significant, *F* < 1.

Two ANOVAs with Group (patients and controls) as the between-participants factor and Performance (incorrect, correct-fast, and correct-slow) and Feedback Condition (informative and uninformative) as the within-participant factors were performed to analyse the VPP and the FRN activities.

The analysis of the VPP (see Table 2 and Fig 3) amplitude showed a significant effect of Group, *F*(1, 42) = 12.08, *p* = .001, *η*^*2*^_*p*_ = .223. Contrary to the prediction of reduced external signal processing caused by a tendency towards internal signals, patients with panic disorder showed enhanced VPP evoked by external signals. The interaction between Group and the within-participant factors were all not significant, *F*s < 1. The absence of any interaction between Group and Feedback Condition or Performance suggests that, despite the enhanced processing of external signals, patients showed a normal flexibility in processing an external signal according to its information content and meaning. Beside the significant main effect of Group, the analysis of the VPP amplitude showed also a significant main effect of Feedback Condition, *F*(1, 42) = 201.78, *p* < .001, *η*^*2*^_*p*_ = .828, and a significant main effect of Performance, *F*(1.17, 48.97) = 4.33, *p* = .037, *η*^*2*^_*p*_ = .093. The interaction between Feedback Condition and Performance was significant, *F*(1.17, 49.14) = 9.34, *p* = .002, *η*^*2*^_*p*_ = .182, as well. This interaction indexed that the VPP was overall more positive in the processing of informative feedback compared to the processing of uninformative feedback. In fact, post-hoc tests showed that the VPP was larger for informative than uninformative feedback both in trials with incorrect responses, *t*(43) = 8.21, *p* < .001, and in trials with correct responses that were fast, *t*(43) = 13.01, *p* < .001, or slow, *t*(43) = 11.68, *p* < .001. However, Performance had a significant effect only when feedback was informative, *F*(1.12, 48.19) = 8.33, *p* = .004, *η*^*2*^_*p*_ = .164, while the effect was not significant in the condition with uninformative feedback, *F*(1.35, 57.98) = 1.30, *p* = .270. Taken together, these outcomes indicate that the VPP is more positive when feedback is meaningful for performance monitoring, suggesting a flexible interpretation of an external signal according to contextual factors. However, the present findings do not clarify whether performance quality affects the VPP, because of the employment of perceptually dissimilar stimuli in the condition with informative feedback.

**Table 2:** Mean amplitudes of the VPP and the FRN.

	VPP		FRN	
	peak-to-peak		area 200–300	
	Patients	Controls	Patients	Controls
**Informative feedback**				
** • error**	16.62 (1.35)	12.57 (1.37)	3.22 (1.15)	2.43 (1.53)
** • correct-fast**	19.09 (1.02)	14.55 (1.09)	5.16 (0.80)	3.88 (1.22)
** • correct-slow**	19.27 (0.93)	14.12 (1.06)	5.01 (0.78)	3.38 (1.28)
**Uninformative feedback**				
** • error**	12.15 (1.05)	7.63 (0.93)	-1.40 (0.92)	-2.49 (1.37)
** • correct-fast**	11.85 (0.92)	7.02 (0.97)	1.81 (0.67)	-0.49 (0.99)
** • correct-slow**	12.10 (0.97)	7.94 (0.92)	1.54 (0.66)	-1.01 (1.03)
**Stimulus**				
** • faces**	17.32 (1.04)	12.19 (0.98)		
** • houses**	10.36 (0.88)	7.28 (0.74)		

Contrary to the significant main effect of Group in the VPP analysis (see Table 2 and Fig 3), patients and controls did not present any significant difference in both the FRN amplitude, *F*(1, 42) = 1.43, *p* = .232, and the FRN modulations induced by Feedback Condition and Performance, *F*s < 1. The absence of any effect of Group on the FRN might suggest that panic disorder does not affect the evaluation of external signals according to their meaning for performance monitoring. Reverting to the within-participant factors, the FRN results showed a significant effect of Feedback Condition, *F*(1, 42) = 104.10, *p* < .001, *η*^*2*^_*p*_ = .713, a significant effect of Performance, *F*(1.17, 49.28) = 15.81, *p* < .001, *η*^*2*^_*p*_ = .273, but no interaction between the within-participant factors, *F*(1.22, 51.05) = 1.27, *p* = .273. On the one hand, the main effect of Feedback Condition reflected overall less negative FRN amplitudes in the condition with informative feedback compared to the condition with uninformative feedback. On the other hand, the significant main effect of Performance reflected more negative FRNs in trials with incorrect responses compared to trials with correct responses that were fast, *t*(43) = 4.63, *p* < .001, or slow, *t*(43) = 3.51, *p* = .001, but no difference between correct trials with fast or slow responses, *t*(43) = 2.12, *p* = .040.

In the employed design, the 500-ms delay between response and feedback onset was not sufficiently long for the complete decline to baseline activity of late response-related potentials, particularly after errors. This problem determined a significant effect of Performance and Feedback Condition in the time-window 0–100 ms after feedback onset, *F*s > 7.14, *p*s < .011. The employment of a shorter baseline (from -50 to 0) was a better correction because it avoided residual response-locked activities and produced ERPs that did not present any significant influence of the within-participant factors in the time-window 0–100 ms, *F*s < 2.52, *p*s > .101.

The statistic performed on the ERPs computed with the 50-ms baseline correction, confirmed the absence of any effect of Group on the FRN, *F*s < 1.49, *p*s > .228. Moreover, the main effects of Performance and Feedback Condition were both significant, *F*(2,41) = 3.70, *p* = .033 and *F*(1,42) = 104.31, *p* < .001, and their interaction was not significant, *F* < 1. Therefore, residual response-locked activity did not confound the FRN results. Baseline problems did not apply to the VPP analysis because of the employment of a peak-to-peak measurement.

#### Passive viewing task

**Fig 4:**
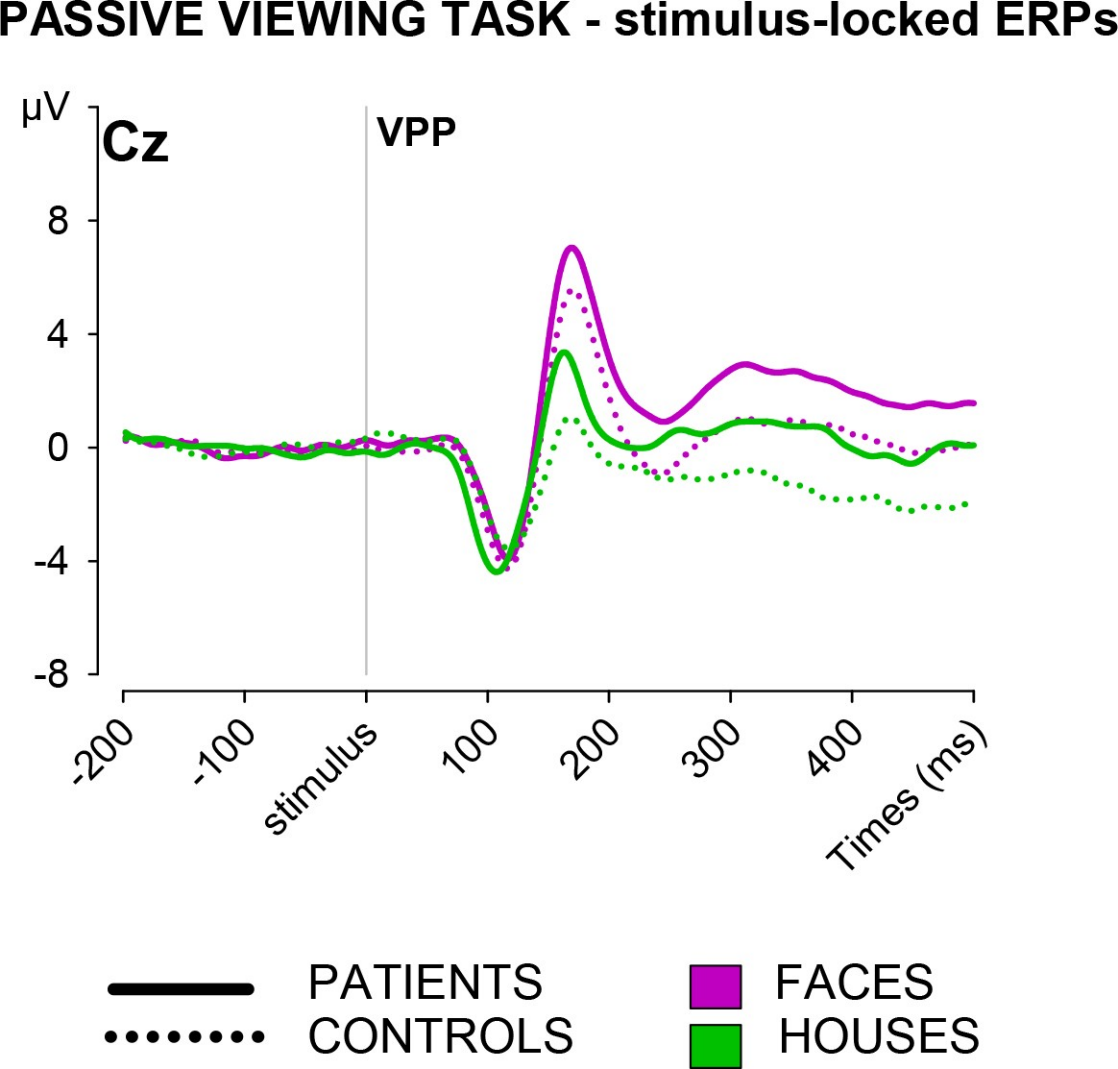
ERP results–passive viewing task. Stimulus-locked grand average ERP waves for the processing of faces and houses.

In the analysis of the VPP potentials evoked by pictures of faces or houses, Group was significant as a main effect, *F*(1, 42) = 12.53, *p* = .001, *η*^*2*^_*p*_ = .230 (see Table 2 and Fig 4). As observed for feedback processing in the response-choice task, patients presented enhanced VPPs. Stimulus was significant as a main effect, *F*(1, 42) = 107.30, *p* < .001, *η*^*2*^_*p*_ = .719, indicating more positive VPPs for faces than houses, but the interaction between Stimulus and Group was short of significance, *F*(1, 42) = 3.21, *p* = .080.

## Discussion

Panic disorder, like other psychological disorders associated with anxiety, depression, or obsessive-compulsive symptoms, is considered a mental illness characterised by a pathological tendency towards the internal world that often leads to withdrawal from the external world. The present experiment investigated whether patients with panic disorder present a deficit in the flexible processing of external signals for performance monitoring. We expected that the enlarged processing of internal signals in panic disorder occurred with a reduced and inflexible processing of external signals. Contrary to the prediction of withdrawal from the external world, ERPs evoked by feedback signals were larger in patients than in controls. Interestingly, augmented processing of external signals in patients was not restricted to feedback in the context of performance monitoring but generalised to pictures of faces and houses in a passive viewing task. Moreover, despite the abnormal processing of external signals in panic disorder, patients and controls showed a similar sensitivity to the feedback information content suggesting a normal attitude to process an external information according to contextual factors.

Many studies of performance monitoring showed that anxiety, depression, and obsessive-compulsive symptoms, both in the clinical and subclinical population, are linked to an enhanced monitoring of errors based on internal signals, indexed by larger Ne/ERN in patients compared to controls (for reviews, see [10, 11, 64]). The present experiment replicates this observation in patients with panic disorder, showing that the abnormal monitoring of errors in internalization is a characteristic feature also of disorders with fear symptoms [1]. As previously observed by Olvet and Hajcak [48], the offer of an additional source for performance monitoring, as in the condition with informative feedback, did not reduce the amplitude of the Ne/ERN in both the patient and the control group. The insensitivity of this ERP to contextual factors indicates that the processing of internal signals evoked by errors is inflexible and that the emotional reaction evoked by errors is not postponed to an external signal when an establish stimulus-response mapping enables the detection of incorrect responses based on internal signals, as in response-choice tasks [40, 41, 43].

Contrary to the stability of the ERPs evoked by internal signals, changing the feedback information content across the experimental runs had significant effects on the amplitude of the ERPs evoked by the external signals. The VPP was more positive for informative than uninformative feedback, and this positive activity was than sustained over the subsequent ERPs. This result replicates previous observations of more negative electrophysiological response in the processing of feedback signals that are not informative about the personal performance because related to the performance of another participant or randomly generated by an algorithm [24, 47]. We can exclude that the observed effect of feedback condition reflects the employment of perceptually different signals between the conditions because the result was significant also for the contrast between scrambled faces used as informative or uninformative feedback of errors. Importantly, the absence of any significant difference in external signal processing according to feedback information content between patients and controls shows that internalization does not significantly influence the capacity to process flexibly an external signal according to its information content. This result contradicts Ingram’s [3] concept of self-absorption, where self-focused attention should hamper the capacity to process external signals accurately.

Moreover, contrary to the theory of self-absorption [3], patients with panic disorder showed enhanced processing of external signals, indexed by the more positive VPP. This result is difficult to reconcile with the idea that a pathological focus towards the internal world subtracts monitoring resources for the accurate processing of the external world. In fact, the enhanced VPP in patients indicates that the allocation of attention to external signals was larger in patients than in controls. Interestingly, the enhanced processing of external signals was evident also in the passive viewing task, showing that the inclination to pay attention to external signals is not circumscribed to feedback processing. These results find a correspondence in the observation that anxiety enhances the allocation of attention to stimuli in a word-emotion Stroop task, irrespective of the emotional content [65]. Despite previous observations of an attentional bias for disorder-related stimuli [66], Fisher and collaborators [67] showed that, in panic disorder, neutral and emotional words evoked an overall larger P200, an ERP evoked over frontal recording regions that peaks at around 200 ms after word onset. The present results support the observation that, in panic disorder, stimulus processing is enhanced regardless of its emotional content because neutral and emotional faces evoked enhanced VPPs with similar amplitudes and this potential was larger also in the processing of pictures of houses or scrambled faces. Therefore, panic disorder seems to be linked to a general heighten vigilance towards external signals, together with a larger processing of internal signals associated with errors. The observed hypervigilance might reflect an increased motivation or engagement in the task, as performing well might be more important in patients.

Interestingly, patients and control participants did not present any significant difference in the pattern of FRN activity [10]. Previous studies on psychological disorders found abnormal processing at the level of the FRN, suggesting a differential evaluation of feedback meaning as reduced rewarding of positive feedback [68] or enhanced sensitivity to negative material [69]. In the present experiment, instead, the absence of any significant effect of group on the FRN seems to indicate that panic disorder does not affect feedback interpretation. However, the present FRN results, particularly of within-subject factors, should be considered with caution because of the use, in one context, of emotional faces as informative feedback of correct-fast and correct-slow responses. In fact, the occurrence of emotional processing in the context with informative feedback might have confounded the accurate analysis of the dynamic of feedback processing [70]. Moreover, the presentation of external signals after errors lacking any negative connotation might have masked the bias of patients towards negative material and the consequent FRN modulations.

The results of the present experiment invite to have a broader perspective on the functional deficits in panic disorder and the potential effects of treatment on abnormal performance monitoring. In fact, neuroimaging studies reported that psychotherapy induces changes towards normalisation of abnormal brain responses (for a review, see [71]). However, electrophysiological studies of performance monitoring suggest that brain functioning does not change after psychotherapy [12, 72, 73]. For example, Kujawa and collaborators [73] reported that, despite a reduction of the symptoms, psychotherapy did not induce any significant decrease of the abnormal amplitude of the Ne/ERN in patients with social anxiety. In sight of the present experiment, psychotherapy might still be effective in normalising other abnormal brain processes, as the enhanced processing of external signals observed in the group of patients with panic disorder.

In conclusion, the present results conflict with the prediction that a pathological tendency towards the internal world hinders the accurate and flexible processing of the external world in panic disorder [3]. This observation does not exclude that withdrawal from the external world, with a consequent reduced and inflexible processing of external signals, occurs in other psychological disorders characterised by internalization. In fact, according to Krueger [1], panic disorder is linked to high fear whereas MDD and GAD are associated with anxiety and misery symptoms. Therefore, according to the present results, fear might induce a hyper-vigilance of the environment for the detection of events that might cause a panic attack. Future experiments should investigate whether a deficit in the processing of external signals is present in MDD or GAD, where the internal expression of distress and misery, with the consequent focus of attention on the internal world, might distract from an accurate and flexible processing of the external world. Moreover, future studies should analyse the relevance of a comorbidity of a personality disorder for the manifestation of abnormal brain processing, particularly in relation to the processing of external signals.
